# Complete Genomic Analysis of a Kingdom-Crossing *Klebsiella variicola* Isolate

**DOI:** 10.3389/fmicb.2018.02428

**Published:** 2018-10-09

**Authors:** Yatao Guo, Yao Zhai, Zhao Zhang, Daixi Li, Zhanwei Wang, Jingquan Li, Zilong He, Songnian Hu, Yu Kang, Zhancheng Gao

**Affiliations:** ^1^Department of Respiratory and Critical Care Medicine, Peking University People’s Hospital, Beijing, China; ^2^University of Technology Sydney, Ultimo, NSW, Australia; ^3^Laboratory Medicine, Peking University People’s Hospital, Beijing, China; ^4^Institute of Botany, Chinese Academy of Sciences, Beijing, China; ^5^Beijing Institute of Genomics, Chinese Academy of Sciences, Beijing, China

**Keywords:** *Klebsiella variicola*, genome, virulence, resistance, endophyte

## Abstract

Bacterial isolate X39 was isolated from a community-acquired pneumonia patient in Beijing, China. A phylogenetic tree based on *rpoB* genes and average nucleotide identity data confirmed that isolate X39 belonged to *Klebsiella variicola*. The genome of *K. variicola* X39 contained one circular chromosome and nine plasmids. Comparative genomic analyses with other *K. variicola* isolates revealed that *K. variicola* X39 contained the most unique genes. Of these unique genes, many were prophages and transposases. Many virulence factors were shared between *K. variicola* X39 and *Klebsiella pneumoniae* F1. The pathogenicity of *K. variicola* X39 was compared with that of *K. pneumoniae* F1 in an abdominal infection model. The results indicated that *K. variicola* X39 was less virulent than typical clinical *K. pneumoniae* F1. The genome of *K. variicola* X39 also contained some genes involved in plant colonization, nitrogen fixation, and defense against oxidative stress. GFP-labeled *K. variicola* X39 could colonize maize as an endophytic bacterium. We concluded that *K. variicola* X39 was a kingdom-crossing strain.

## Introduction

The *Klebsiella* genus consists of pathogens capable of colonizing and infecting humans and animals, as well as bacteria capable of colonizing plants as endophytes (Podschun and Ullmann, 1998). As the leading *Klebsiella* pathogen, *Klebsiella pneumoniae* is one of the most common pathogens of hospital-acquired pneumonia (Chung et al., 2011). Nevertheless, with the development of sequencing technology and molecular epidemiology, methods of phylogenetic reconstruction can divide *K. pneumoniae* into three distinct *Klebsiella* species: *K. pneumoniae*, *K. quasipneumoniae*, and *K. variicola* (Rosenblueth et al., 2004; Brisse et al., 2014; Maatallah et al., 2014). *K. variicola* was first proposed as a new *Klebsiella* species in 2004 based on clinical and plant-associated isolates (Rosenblueth et al., 2004). The species *K. singaporensis* was first isolated from a single soil isolate in 2004 (Li et al., 2004) and identified as a junior heterotypic synonym of *K. variicola* (Brisse et al., 2014). *K. variicola* is able to fix N_2_ and is abundant in the environment. As the current VITEK automated system is unable to efficiently distinguish the *Klebsiella* species, some of the clinical *Klebsiella* isolates previously considered *Klebsiella pneumoniae* may in fact be *Klebsiella variicola* or *K. quasipneumoniae*. For example, former *K. pneumoniae* 342 was identified as an isolate of *K. variicola* (Brisse et al., 2014). *K. pneumoniae* KPC-142 originally reported as *K. pneumoniae* by VITEK 2 system was actually *K. quasipneumoniae subsp. similipneumoniae* KPC-142 (Nicolas et al., 2018). Most of the *K. variicola* strains currently reported were found in the environment, and case reports of *K. variicola* infection were rare. The potential for clinical *K. variicola* isolates to colonize plants is poorly understood, and the mechanism of plant-associated *Klebsiella* isolates as drug-resistance genes reservoirs remains unclear.

In the present study, *K. variicola* X39 was isolated from the sputum of a community-acquired pneumonia patient and revealed resistance to a variety of antibiotics. We compared the pathogenicity of *K. variicola* X39 with that of typical clinical *K. pneumoniae* F1. On the other hand, we confirmed that *K. variicola* X39 was able to colonize plant roots, stems, and leaves by labeling *K. variicola* X39 with green fluorescent protein (GFP). Here, we analyzed the genomic characteristics of *K. variicola* X39 and laid the foundation for understanding the resistance, virulence, endophytic lifestyle of *K. variicola*.

## Materials and Methods

### Bacterial Isolation, Clinical Information, Biochemical Identification, and Antimicrobial Susceptibility Testing

An acute renal failure patient with pulmonary infection was admitted to the Department of Nephrology, Peking University People’s Hospital in March 2011. *K. pneumoniae* (eventually identified as *K. variicola*) was cultured three times consecutively in the sputum of the patient. Written informed consent was obtained from the patient. The isolate was designated X39 for further study. The patient eventually died of lung infection (**Supplementary Figure S1**) and heart failure. Biochemical identification of isolate X39 was determined using the VITEK 2 system and API 20E biochemical assays (bioMérieux, Marcy-l’Étoile, France). The antimicrobial susceptibilities of isolate X39 were determined by identifying the minimum inhibitory concentration (MIC) values by the VITEK 2 system, in accordance with the guidelines of the Clinical and Laboratory Standards Institute.

### Genome Sequencing and Assembly

*Klebsiella variicola* X39 was cultured overnight in Luria–Bertani broth at 37°C. We used a QIAamp DNA Mini Kit (QIAGEN, Hilden, Germany) to extract the genomic DNA and determined the quality and concentration of DNA using a NanoDrop spectrometer (Thermo Scientific, Wilmington, MA, United States). A 4- to 10-kb insert library was obtained from the genomic DNA of *K. variicola* X39 and sequenced by Pacific Bioscience’s (PacBio, Menlo Park, CA, United States) Single Molecule, Real-Time (SMRT) sequencing technology at the Beijing Institute of Genomics, Chinese Academy of Sciences. Sequencing was performed on two SMRT cells, and average coverage was 84×. The pacific sequencing yielded 201,794 high-quality filtered reads with an average length of 3,170 bp, and the N50 read length was 4,556 bp. Hierarchical genome assembly process (HGAP) with SMRT analysis 3.0 was used to assemble the high-quality reads. The preliminary assembly results obtained were compared and analyzed. The chromosomal and plasmid sequences were screened and, respectively, assembled into circular DNA, i.e., the final 0 gap complete sequence.

### Genome Annotation

GeneMarkS software was used to predict the protein-encoding genes (Besemer et al., 2001). The GO, KEGG, COG, and NR annotation of each predicted gene was assigned based on the results of BLASTP (E-value ≤10^-5^; identity ≥40%; coverage ≥40%). TRNAscan-SE version 1.3.1 (Lowe and Eddy, 1997), rRNAmmer version 1.2 (Lagesen et al., 2007), and Rfam (database version 11) (Gardner et al., 2009; Nawrocki et al., 2009) were used to predict the tRNAs, rRNAs, and sRNAs, respectively. Genomic islands (GIs), prophages, and CRISPRS were predicted by IslandPath DIMOB (Hsiao et al., 2005), PHAST (database download date: November 10, 2014) (Zhou et al., 2011), and CRISPRFinder^1^ (Grissa et al., 2007).

### Phylogenetic Analyses and Average Nucleotide Identity Analysis

The complete nucleotide sequence of *rpoB* genes from other *K. pneumoniae*, *K. quasipneumoniae*, and *K. variicola* isolates (**Supplementary Table S1**) was downloaded from the GenBank database. The *rpoB* genes from *Klebsiella oxytoca* CAV1374 (CP011636.1), *Klebsiella oxytoca* KONIH1 (CP008788.1), and *Escherichia coli* K-12-MG1655 (U00096.3) were also included in the phylogenetic analysis. The phylogenetic tree was constructed using PHYML (maximum likelihood, ML) with a Tamura-Nei parameter model and 1,000 bootstrap replications (Mega X) (Kumar et al., 2018). Average nucleotide identity analysis was performed between *K. variicola* X39 and other *Klebsiella* isolates (**Supplementary Table S1**) included in the phylogenetic tree using an online ANI Calculator^2^ (Yoon et al., 2017).

### Comparative Genomic Analyses

*Klebsiella variicola* X39 was clinically isolated. We further selected two non-clinical *K. variicola* isolates - *K. variicola* 342 (Brisse et al., 2014) and *K. variicola* DX120E (Lin et al., 2015) - and two clinical *K. variicola* isolates - *K. variicola* MGH20 and *K. variicola* MGH40 - whose genome sequences were available in GenBank. The homologous genes were calculated using Cd hit (v4.6.1) with a threshold of default 0.5 (Li and Godzik, 2006). Homologous genes present in all five isolates served as core genes. After removing the core genes, we obtained the unique genes of each isolate.

### Pathogenicity Testing

In order to evaluate the pathogenicity of *K. variicola* X39, we generated an abdominal infection animal model. This study was performed in accordance with the principles of the Basel Declaration and recommendations of the National Laboratory Animal Standardization Technical Committee. The protocol was approved by the Peking University People’s Hospital Institutional Animal Care and Use Committee (2015–27). *K. pneumoniae* isolate F1 was a clinical strain isolated from a patient with urinary tract infection in our laboratory, where we sequenced its genome (CP026130.1). *K. pneumoniae* isolate F1 was also included in the study. Eight-week-old female BALB/c mice were obtained from Sibeifu (Beijing, China) Laboratory Animal Science and Technology Co., Ltd. The overnight culture of *K. variicola* X39 and *K. pneumoniae* F1 were washed by saline and resuspended in saline. 5 × 10^9^, 5 × 10^8^, 5 × 10^7^, and 5 × 10^6^ CFU/ml were obtained by serial dilution to determine the median lethal dosage (LD50). Six mice were injected at each concentration. Each BALB/c mouse was injected 200 μL bacterial suspension intraperitoneally. Six mice were injected with 200 μL saline each mouse as a negative control group. All mice were monitored daily for survival for 14 days. The LD50 was calculated using the probit regression model of SPSS 24.

### *K. variicola* X39 Competent Cells

The competent cells of *K. variicola* X39 were prepared as follows: frozen *K. variicola* X39 was inoculated on a blood agar plate and cultured overnight at 37°C. The isolates were then inoculated in 15 mL precooled ultrapure water and immediately centrifuged at 5,000 rpm for 5 min at 4°C. The supernatant was removed and discarded, and the cell pellet was resuspended in 15 mL precooled ultrapure water. These steps were repeated twice. The density of bacteria was measured by the WGZ-2XJ Bacteria Turbidity Meter (Shanghai Xin Rui, Shanghai, China). The bacterial turbidity was finally concentrated to 3, and the suspension was divided into 100-μL tubes.

### Construction of GFP-Labeled *K. variicola* X39

Vector plasmid puA66 was provided by Professor Uri Alon, which contained GFP and kanamycin-resistance gene *kan* (**Figure 4A**). A CTX-M-14 gene promoter was inserted upstream of the GFP open reading frame in the pUA66 plasmid so that the fluorescence intensity of GFP could be directly observed by fluorescence microscopy (Wang et al., 2014). Purified plasmid DNA (1 μg) was added to 100-μL ice-chilled cell suspension and mixed gently. The mixture was bathed in ice for 30 min and then transferred to an ice-chilled electroporation cuvette (1 mm electrode gap, Bio-Rad, Richmond, CA, United States). Electroporation was performed using the Bio-Rad Micropublisher apparatus. Pulse voltage and pulse time were 2.1 kV and 4 ms, respectively. Luria–Bertani medium (900 μL) was added to the electroporation cuvette and mixed gently. The bacterial solution was transferred to a 15-mL sterile centrifuge tube and shaken for 1 h at 190 rpm. The bacterial solution (100 μL) was plated on Mueller–Hinton Agar medium containing 100 mg/mL kanamycin. Colonies were inoculated in 1 mL Luria–Bertani medium, shaken for 3 h, and then observed under an Olympus FV1000MPE multiphoton laser scanning microscope (Tokyo, Japan).

### Plant Growth and Inoculation

Maize seeds were soaked in 1% sodium hypochlorite solution for 10 min and then in 75% alcohol for 5 min to kill bacteria on the surface of seeds. Next, the seeds were rinsed repeatedly with sterile water. Then, the seeds were placed on wet sterile papers in a sterile bottle at room temperature, and after about 3 days, the maize roots were approximately 2 cm in length. The germinated seedlings were transferred to a sterile tissue culture bottle (10.8 cm in height and 7.5 cm in diameter) filled with 20 mL 1/2 × Murashige & Skoog medium (Murashige and Skoog, 1962). Next, 2 mL GFP-labeled *K. variicola* X39 cells (1 × 10^9^ cells/mL) were added to the tissue culture bottle. Phosphate-buffered saline solution (2 mL) was added to the control group.

### Colonization of GFP-Labeled *K. variicola* X39 in Maize

The maize seedlings were retrieved from the 1/2 Murashige & Skoog medium after 3 and 10 days, respectively. The maize root surfaces were rinsed with sterile water and then directly observed under the Olympus FV1000MPE multiphoton laser scanning microscope. The stems were sectioned in the transverse direction, and the leaves and epidermis were separated to observe the colonization of GFP-labeled *K. variicola* X39.

## Results

### Genomic Features of *K. variicola* X39

**FIGURE 1:**
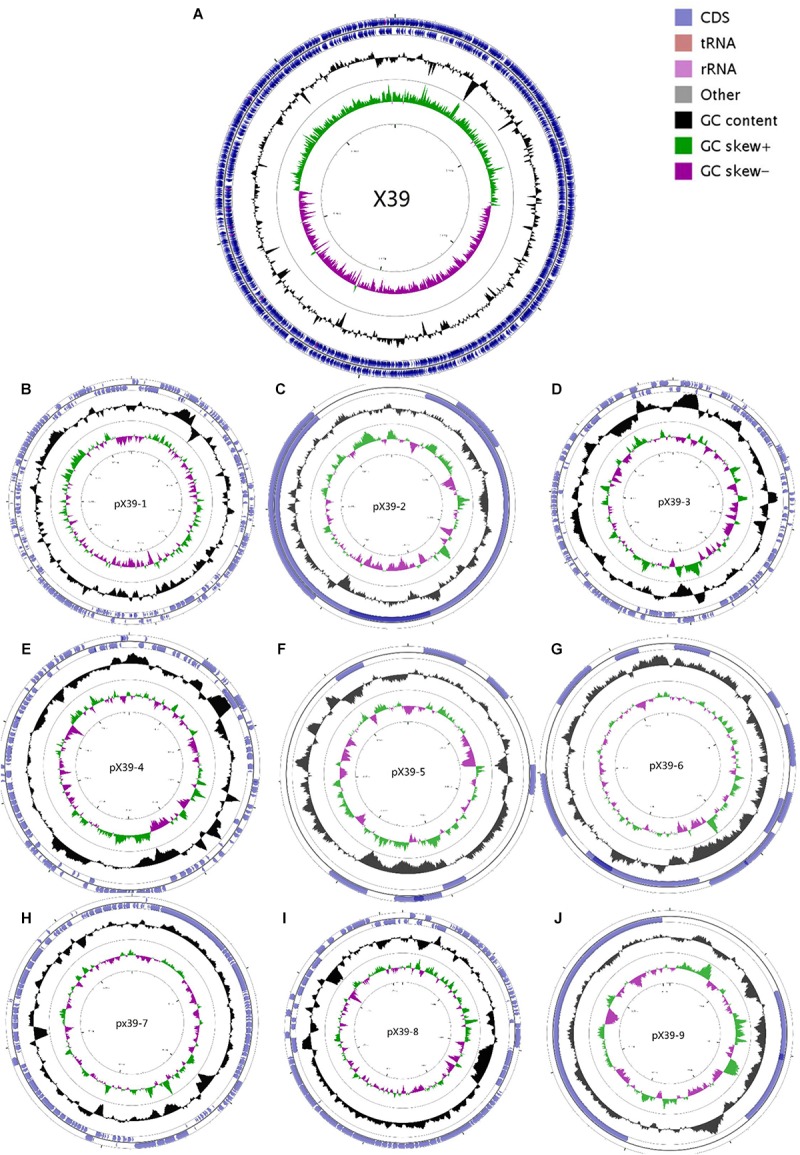
Circular maps of the chromosome **(A)** and nine plasmids **(B–J)** of *K. variicola* isolate X39. From the outside to the center: Genes on the forward strand, genes on the reverse strand, GC content, GC skew. The circular maps were generated by CGView v1.0.

We assembled the genome sequences of isolate X39 into 10 circular replicons: a 5,641,443-bp chromosome and nine plasmids (**Figure 1**). The genomic traits of isolate X39 were provided in **Table 1**. A total of 18 GIs were detected ranging in size from 4528 to 36,309 bp in *K. variicola* X39 (**Supplementary Table S2**). GIs005 was related to type IV and type VI secretion system.

**Table 1:** The genomic traits of *K. variicola* X39.

Trait	Chromosome	pX39-1	pX39-2	pX39-3	pX39-4	pX39-5	pX39-6	pX39-7	pX39-8	pX39-9	Combined
Size (bp)	5,641,443	213,341	3,358	52,398	49,735	3,374	5,399	111,425	72,224	1,431	6,154,128
G + C content (%)	57.30	51	51.70	47.20	52.40	46.20	50.20	49	52.50	51.90	56.78
Open reading frame numbers	5,415	222	3	97	84	2	8	120	86	1	6,038
CRISPRs	4	0	0	0	0	0	0	0	0	0	4
Prophages	6	4	0	1	0	0	0	0	1	0	12
Genomic islands	15	3	0	0	0	0	0	0	0	0	18


**FIGURE 2:**
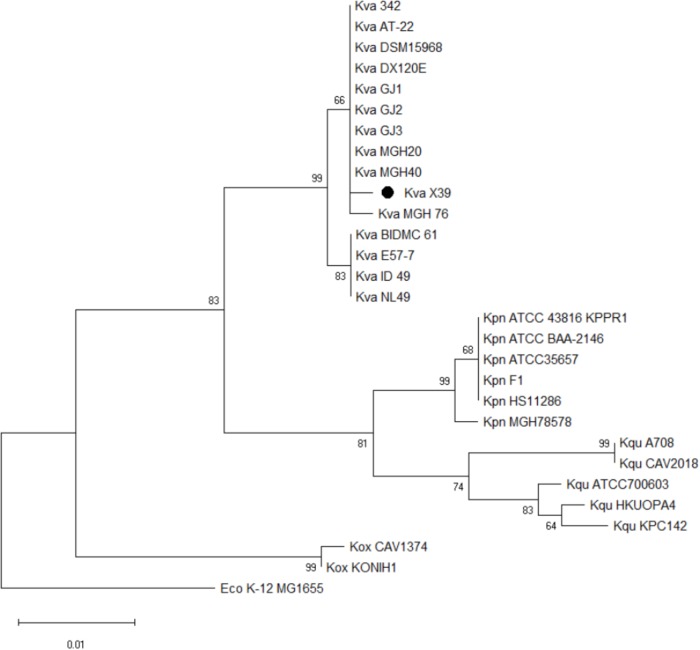
Phylogenetic tree based on *rpoB* genes shows the phylogenetic relationship of isolate X39 (∙) and indicates that it belongs to *Klebsiella variicola*. *Escherichia coli* K-12 MG1655 is used as an outgroup.

We first used the Basic Local Alignment Search Tool (BLAST)^3^ to evaluate the genome of isolate X39. The results indicated that isolate X39 chromosome was most similar to *K. variicola* At-22 chromosome with a 99% identity of 90% coverage. The phylogenetic tree based on the *rpoB* genes showed that *K. pneumoniae* and *K. variicola* were clearly separated, and isolate X39 was closely related to *K. variicola* (**Figure 2**). The genome of *K. variicola* X39 has high average nucleotide identities (Goris et al., 2007) above 98% to the available genomes of *K. variicola* 342, At-22 (Pinto-Tomas et al., 2009), DSM15968, DE120E, GJ1,GJ2,GJ3 (Di et al., 2017), MGH20, MGH40, BIDMC61, E57-7, ID_49, NL49 (**Supplementary Table S3**), and thus belongs to *K. variicola*.

The largest of the nine plasmids, pX39-1, was most similar to plasmid pNH25.1 of *K. pneumoniae* strain NH25 with a 99% identity of 50% coverage. The similar regions mainly encoded transposases/recombinases and ABC transporter permease. The plasmid pX39-4 was most similar to plasmid pEcNDM1 of *E. coli* strain EcNDM1 with a 99% identity of 96% coverage. This similarity was restricted to regions of the plasmids conferring partitioning, conjugative transfer, and replication. The plasmid pX39-8 was most similar to plasmid pE20-qnrS of *Klebsiella aerogenes* strain E20 with a 99% identity of 87% coverage. The similar regions mainly encoded proteins for plasmid conjugative transfer.

We carried out the functional analysis by Cluster of Orthologous Groups (COGS) (**Supplementary Table S4**). Of the 5,812 protein-coding genes, 4,082 genes (70.2%) pertained to a COG category. The results revealed that the three major metabolism classes - amino acid transport and metabolism (E), carbohydrate transport and metabolism (G), and inorganic ion transport and metabolism (P) - represented 25.24% of all protein-coding genes. The high proportion of these genes in the genome indicated *K. variicola* X39 had the inherent potential for efficient uptake of nutrients and competition with surrounding microorganisms (Niazi et al., 2014).

### Genomic Features of *K. variicola* X39 Adaptation to Plants

*Klebsiella variicola* is often found in plants (Rosenblueth et al., 2004; Lin et al., 2015; Martinez-Romero et al., 2015). A high number of genes that were involved in plant colonization, nitrogen fixation, and defense against oxidative stress were found in the genome of *K. variicola* X39. Many plant-associated bacteria could produce cellulose to promote adhesion and colonization of plant roots (Romling and Galperin, 2015). The genome of *K. variicola* X39 contains all genes that can synthesize cellulose (*bcsABCD*) (X39GM004945–X39GM004948). Nitrogen is one of the indispensable micronutrients for plant growth. *K. variicola* has been reported to fix nitrogen since it was discovered (Rosenblueth et al., 2004). *K. variicola* X39 contains all genes encoding the nitrogenase enzyme (*nifDHK*) (X39GM001181–X39GM001183). After contact with bacteria, plants can produce a range of defense substances including phytoalexins, nitric oxide, and reactive oxygen (Zeidler et al., 2004). Therefore, endophytes must be able to tolerate the reactive oxygen environment during colonization. Accordingly, a series of enzymes and regulatory factors that can help bacteria deal with oxidative stress were found in the genome of *K. variicola* X39, including superoxide dismutase (*sod*, X39GM001732, X39GM001742, and X39GM004640), catalase (*katG*, X39GM001916; *katE*, X39GM002512; *katN*, X39GM002637), 10 putative peroxidases, three hydroperoxide reductases (X39GM00328, X39GM003285, and X39GM003608), and 15 putative glutathione S-transferases or glutathione S-transferase domain/family proteins. An AcrAB (X39GM003513-3514) locus, belonging to the RND transporter family and necessary for the export of apple tree phytoalexins and the successful colonization of host plants (Burse et al., 2004), was also identified in the genome of *K. variicola* X39.

### Comparative Genomic Analysis

**FIGURE 3:**
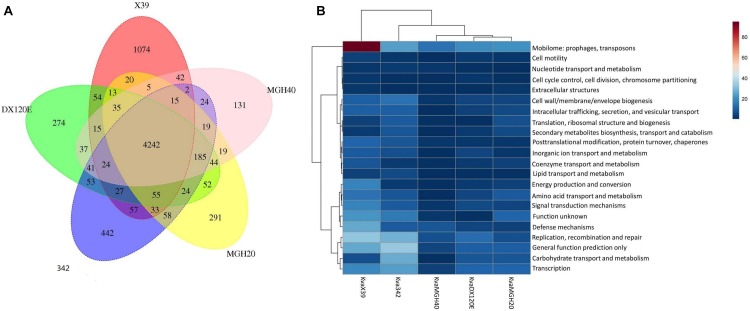
**(A)** Whole-genome comparison of *K. variicola* X39, *K. variicola* MGH20, *K. variicola* MGH40, *K. variicola* DX120E, and *K. variicola* 342. **(B)** The heatmap of the COG category of the unique genes in *K. variicola* X39, *K. variicola* MGH20, *K. variicola* MGH40, *K. variicola* DX120E, and *K. variicola* 342. The heatmap was made using MetaboAnalyst 4.0.

The putative orthology between *K. variicola* X39 and the other four *K. variicola* isolates were determined by comparative genomic analysis. These results revealed that 4,242 gene clusters were shared between them. *K. variicola* X39 contained the most unique gene clusters (**Figure 3A**). A total of 1,074 gene clusters were identified as exclusive to *K. variicola* X39 (**Supplementary Table S5**). Of these unique gene clusters, 550 genes were from the nine plasmids, and 588 genes were from the chromosome. The COG category of the unique genes showed that *K. variicola* X39 possessed more genes related to energy production and conversion, amino acid transport and metabolism, coenzyme transport and metabolism, signal transduction mechanisms, defense mechanisms, and mobilome: prophages, transposons compared with other four *K. variicola* isolates (**Figure 3B** and **Supplementary Table S6**). *K. variicola* X39 possessed at least 37 transposases or putative transposases not found in the other four *K. variicola* isolates. *K. variicola* X39 and the other four *K. variicola* isolates differed in the distribution of genes encoding type IV and type VI secretion systems. Eight type IV secretion proteins and six type VI secretion proteins identified in *K. variicola* X39 were absent in the other four *K. variicola* isolates (**Supplementary Table S5**).

### Virulence of *K. variicola* X39

We compared the genome of *K. variicola* X39 with the Virulence Factors of Pathogenic Bacteria Database (VFDB 2012 update) and identified 155 virulence factors. These virulence factors mainly included some genes related to adhesion, capsule production, and iron acquisition. Siderophores, such as IroN (X39GM002504), aerobactin (X39GM002739), and enterobactin (*entABCDEF* and *fepABCDG*), were extensively distributed among *K. variicola* X39. Simultaneously, we compared the virulence factors of *K. variicola* X39 with those of typical clinical *K. pneumoniae* F1 (**Supplementary Figure S2** and **Supplementary Table S7**). The results indicated that *K. variicola* X39 and *K. pneumoniae* F1 shared many virulence factors.

### Antibiotic Resistance

*Klebsiella variicola* X39 showed resistance to many antibiotics (**Table 2**). A series of efflux pumps and β-lactamase genes conferring resistance to a variety of drugs were found in the genome of *K. variicola* X39. The genome encoded three β-lactamase genes (X39GM000612, X39GM001420, and X39GM002154). Of these, both X39GM000612 and X39GM001420 encode class A extended-spectrum β-lactamase CTX-M-24. X39GM000612 was located in pX39-8, and X39GM001420 was located in the chromosome. *Len* (X39GM002154) is an intrinsic resistance gene of *K. variicola*. *Qnrs1* (X39GM000620), which was first identified in a clinical isolate of *Shigella flexneri* 2b in Japan (Hata et al., 2005), was located in pX39-8 and conferred a plasmid-mediated quinolone resistance (Martínez-Martínez et al., 1998). *K. variicola* X39 also included two efflux pump superfamilies: the major facilitator superfamily, including the RosA/B efflux pump, and the resistance nodulation division superfamily, including the AcrAB–TolC multidrug efflux pump. The RosA/B efflux pump was first reported in *Yersinia* conferring resistance to cationic antimicrobial peptides (CAMPs) (Bengoechea and Skurnik, 2000). The AcrAB–TolC efflux pump conferred resistance to aminoglycosides, glycylcyclines, macrolides, β-lactams, and acriflavine.

**Table 2:** *K. variicola* X39 antibiotic-resistance profile.

Drug family	Drug	MIC (μg/ml)	Phenotype
Aminoglycoside	Amikacin	≤2	Sensitive
	Gentamicin	≤1	Sensitive
	Tobramycin	8	Intermediate
Quinolone	Ciprofloxacin	≥4	Resistant
	Levofloxacin	2	Sensitive
β-Lactam	Ampicillin	≥32	Resistant
	Ampicillin/sulbactam	≥32	Resistant
	Piperacillin	≥128	Resistant
	Piperacillin/tazobactam	8	Sensitive
	Cefazolin	≥64	Resistant
	Cefuroxime	≥64	Resistant
	Cefuroxime axetil	≥64	Resistant
	Cefotetan	≤4	Sensitive
	Ceftazidime	16	Resistant
	Ceftriaxone	≥64	Resistant
	Cefepime	4	Intermediate
	Aztreonam	≥64	Resistant
	Imipenem	≤1	Sensitive
	Meropenem	≤0.25	Sensitive
Sulfonamide	Trimethoprim/sulfamethoxazole	≥320	Resistant
Others	Furantoin	≥512	Resistant


### Pathogenicity of *K. variicola* X39

We used the BALB/c mice to study the pathogenicity of *K. variicola* X39. Clinical *K. pneumoniae* isolate F1 was used as a control group. *K. variicola* X39 showed an LD50 of 1.97 × 10^7^ CFU in the BALB/c mice, and the LD50 of *K. pneumoniae* F1 was 7.36 × 10^6^ CFU. The negative control group survived until sacrificed.

### Construction of GFP-Labeled *K. variicola* X39 and Colonization in Maize

**FIGURE 4:**
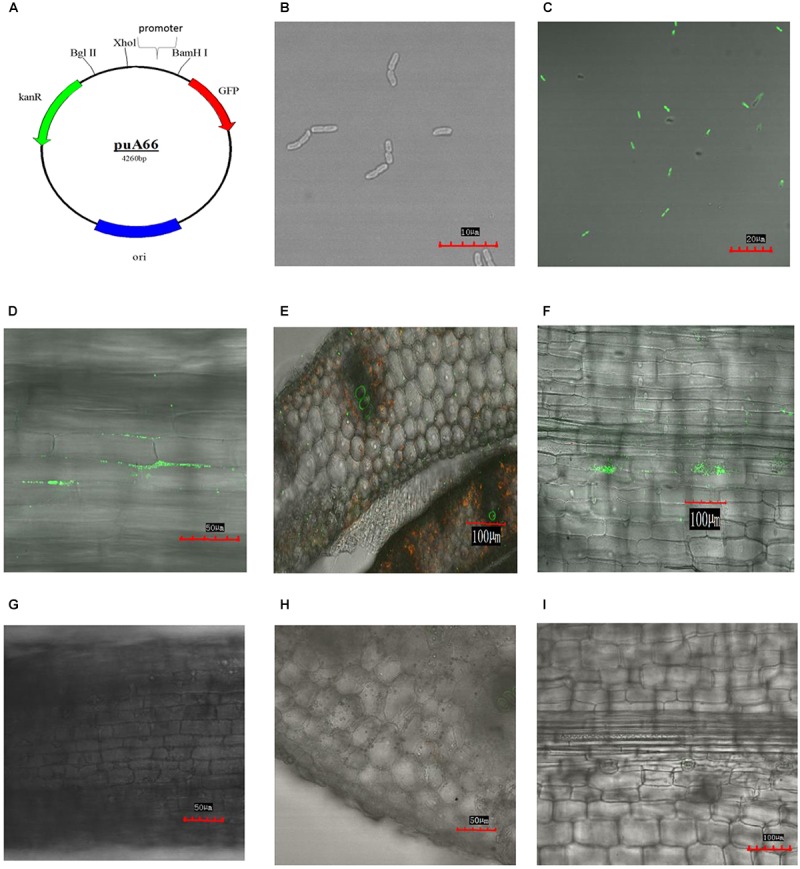
**(A)** Construction of plasmid puA66. **(B)** Confocal image of *K. variicola* X39. **(C)** Confocal image of GFP-labeled *K. variicola* X39. **(D)** Colonization patterns in root after 3 days of inoculation. **(E)** Colonization patterns in stem after 10 days of inoculation. **(F)** Colonization patterns in leaf after 10 days of inoculation. **(G–I)** Confocal images of the respective control groups.

*Klebsiella variicola* X39 itself does not produce green fluorescence (**Figure 4B**). In order to visually observe whether *K. variicola* X39 can colonize plants, we transfected *K. variicola* X39 with a reconstructive plasmid pUA66 that produces GFP (**Figure 4C**). The GFP-labeled *K. variicola* X39 was used to inoculate maize seedlings grown in the gnotobiotic system. Three days after inoculation, GFP-labeled *K. variicola* X39 cells were found to be attached to the root surface and distributed within the cortex (**Figure 4D**). Ten days after inoculation, transverse sections showed that bacterial cells were distributed inside the stems (**Figure 4E**). A large number of bacterial cells were also found in leaves (**Figure 4F**). No bacteria that produced GFP were found in the control group (**Figures 4G–I**).

## Discussion

*Klebsiella variicola* was firstly identified in 2004 in Mexico and consisted of plant isolates and clinical isolates (Rosenblueth et al., 2004). For many years, *K. variicola* was viewed most commonly as a benign endosymbiont of plants or occasionally an opportunistic pathogen. In our study, we identified isolate X39, which was initially identified as *K. pneumoniae* by the VITEK 2 system, from the sputum of a community-acquired pneumonia patient. We used PacBio sequencing technology to obtain the whole-genome sequence of isolate X39. Martinez-Romero et al. (2018) used both phylogenetic tree based on *rpoB* genes and average nucleotide identity to identify a lot of misclassified *Klebsiella* spp. genomes. A phylogenetic tree based on *rpoB* genes and average nucleotide identity data confirmed that isolate X39 belonged to *K. variicola*.

We compared the virulence factors of *K. variicola* X39 with those of typical clinical *K. pneumoniae* F1 and found that most virulence factors were common between them. Genomic analysis indicated that *K. pneumoniae* and *K. variicola* shared some virulence determinants that were able to cause infections in humans (Andrade et al., 2014; Garza-Ramos et al., 2015a). The pathogenicity of *K. variicola* X39 was compared with that of *K. pneumoniae* F1 in an abdominal infection model. The result displayed that the LD50 of *K. variicola* X39 was higher than clinical *K. pneumoniae* F1, which indicated that typical clinical *K. pneumoniae* F1 was more virulent than *K. variicola* X39. The result was also consistent with the patterns of *K. variicola* 342 (Fouts et al., 2008). However, another report suggested that *K. variicola* could cause serious infections including bacteremia and could induce higher mortality compared with *K. pneumoniae* (Maatallah et al., 2014).

*Klebsiella variicola* X39 was mainly resistant to quinolone and β-lactam antibiotics. *K. variicola* X39 contained chromosome-encoded CTX-M-24 and plasmid-encoded CTX-M-24, which were very rare in *Enterobacteriaceae*. *Qnrs1*, located in pX39-8, confers a plasmid-mediated quinolone resistance. As far as we know, pX39-8 is the first detected plasmid carrying a *qnrs1* gene conferring quinolone resistance in *K. variicola* isolates of human origin.

*Klebsiella variicola* is mainly found in the environment, especially in plants. We identified many genes that were involved in plant colonization, nitrogen fixation, and defense against oxidative stress in the genome of *K. variicola* X39. Endophytes can promote plant growth through nitrogen fixation; the nitrogen fixation capacity of *K. variicola* X39 was confirmed by acetylene reduction activity (data not shown). We further used the GFP-labeled *K. variicola* X39 to inoculate seedlings of maize, which is one of the most common crops in northern China. We visually observed obvious colonization in maize roots, stems, and leaves three days and 10 days after inoculation, which illustrated that clinical *K. variicola* X39 retained the capacity to colonize plants. Comparative genomic analysis between *K. variicola* X39 and the other *K. variicola* isolates revealed that *K. variicola* X39 contained the most unique genes, many of which were prophages and transposases. Prophages can assist the transfer of antimicrobial resistance genes and virulent factors to bacteria genome (Kutateladze and Adamia, 2010). Transposases are mobile genetic elements that are important for bacteria to adapt to the surrounding environment (Vigil-Stenman et al., 2017). These results may explain why *K. variicola* X39 can adapt to different hosts.

The species name *K. variicola* means “from different sources,” and recent reports confirmed the rationality of the name (Rosenblueth et al., 2004; Andrade et al., 2014; Davidson et al., 2015; Lin et al., 2015). Through our study, we concluded that *K. variicola* X39 was a kingdom-crossing bacterium (van Baarlen et al., 2007) that could infect humans as a pathogen and colonize plants as an endophyte. Although *K. variicola* can fix nitrogen, it should not be used as a biological fertilizer because of its potential pathogenicity. IMI-2 and OXA-181 carbapenemase and New Delhi metallo-β-lactamase have appeared in *K. variicola* isolates in Europe and in an environmental *K. variicola* isolate from an urban river in South Korea, respectively (Zurfluh et al., 2015; Di et al., 2017; Hopkins et al., 2017). Plants can serve as a reservoir for *K. variicola* isolates that were able to opportunistically infect humans or animals, allowing *K. variicola* to exchange genetic information with other environmental pathogens and gain the advantage of selection, which may also be the reason for the increasing resistance of *K*. *variicola*. Recently, a colistin-resistant hypervirulent *K. variicola* isolate has been isolated from the blood of a patient in Sichuan, China (Lu et al., 2018). The plasmid BLAST results implied that isolate X39 may exchange genes with *Enterobacteriaceae*. It is difficult to distinguish *K. variicola* from other *Klebsiella* species using the current VITEK automated system. Some simple molecular biology methods have been developed to identify *K. variicola* (Garza-Ramos et al., 2015b; Fonseca et al., 2017). As more *K. variicola* isolates are identified, the epidemiological characteristics of *K. variicola* will require further studies.

## Data Availability

The sequences of the chromosome and nine plasmids of *K. variicola* X39 have been deposited in GenBank under accession numbers CP018307 and CP023978–CP023986, respectively.

## Author Contributions

ZG, YK, and SH designed the experiments. YG, YZ, ZZ, and DL performed the experiments. ZW collected and identified the isolate. YG and ZH performed isolate sequencing and bioinformatics analysis. JL operated the Olympus FV1000MPE Multiphoton Laser Scanning Microscope. YG wrote the manuscript. All authors reviewed the manuscript and agreed to the publication of this manuscript.

## Conflict of Interest Statement

The authors declare that the research was conducted in the absence of any commercial or financial relationships that could be construed as a potential conflict of interest.

## Footnotes

1 http://crispr.i2bc.paris-saclay.fr

2 https://www.ezbiocloud.net/tools/ani

3 https://blast.ncbi.nlm.nih.gov/Blast.cgi
