# Supplementary material for: Complete Genomic Analysis of a Kingdom-Crossing Klebsiella variicola Isolate
Source: Front Microbiol. 2018 Oct 9;9:2428. doi: 10.3389/fmicb.2018.02428 (PMC6189331; doi:10.3389/fmicb.2018.02428)
Supplement: Supplementary file 3 [file Table_3.docx]

**Table S3.** The average nucleotide identity of *K. variicola* X39 with other *Klebsiella* isolates.

| Isolate name | *K. variicola* X39 |
| --- | --- |
| *K. pneumoniae ATCC35657* | 94.60% |
| *K. pneumoniae F1* | 94.57% |
| *K. pneumoniae MGH .78578* | 94.65% |
| *K. pneumoniae ATCC BAA-2146* | 94.50% |
| *K. pneumoniae ATCC 43816 KPPR1* | 94.53% |
| *K. pneumoniae HS11286* | 94.64% |
| *K. variicola 342* | 98.95% |
| *K. variicola At-22* | 99.06% |
| *K. variicola DX120E* | 99.02% |
| *K. variicola DSM 15968* | 99.10% |
| *K. variicola GJ1* | 99.10% |
| *K. variicola GJ2* | 99.09% |
| *K. variicola GJ3* | 99.09% |
| *K. variicola* MGH20 | 99.15% |
| *K. variicola* MGH40 | 99.05% |
| *K. variicola* MGH76 | 99.06% |
| *K. variicola* BIDMC61 | 99.07% |
| *K. variicola* E57-7 | 99.13% |
| *K. variicola* ID_49 | 98.96% |
| *K. variicola* NL49 | 99.18% |
| *K. quasipneumoniae* ATCC700603 | 93.42% |
| *K. quasipneumoniae* A708 | 93.47% |
| *K. quasipneumoniae* CAV2018 | 93.36% |
| *K. quasipneumoniae* HKUOPA4 | 93.59% |
| *K. quasipneumoniae* KPC142 | 93.48% |
