# Supplementary material for: Complete Genomic Analysis of a Kingdom-Crossing Klebsiella variicola Isolate
Source: Front Microbiol. 2018 Oct 9;9:2428. doi: 10.3389/fmicb.2018.02428 (PMC6189331; doi:10.3389/fmicb.2018.02428)
Supplement: Supplementary file 4 [file Table_4.docx]

**Table S4.** The functional cluster of orthologous genes (COG) classification of predicted genes in *K. variicola* X39.

| COG functional class | CDS | % of CDS |
| --- | --- | --- |
| **Metabolism** |  |  |
| C-Energy production and conversion | 316 | 5.44 |
| E-Amino acid transport and metabolism | 584 | 10.05 |
| F-Nucleotide transport and metabolism | 87 | 1.5 |
| G-Carbohydrate transport and metabolism | 499 | 8.59 |
| H-Coenzyme transport and metabolism | 188 | 3.23 |
| I-Lipid transport and metabolism | 127 | 2.19 |
| P-Inorganic ion transport and metabolism | 384 | 6.6 |
| Q-Secondary metabolites biosynthesis, transport and catabolism | 120 | 2.06 |
| **Cellular processes and signaling** |  |  |
| D-Cell cycle control, cell division, chromosome partitioning | 38 | 0.65 |
| M-Cell wall/membrane/envelope biogenesis | 218 | 3.75 |
| N-Cell motility | 46 | 0.79 |
| O-Posttranslational modification, protein turnover, chaperones | 154 | 2.65 |
| T-Signal transduction mechanisms | 163 | 2.8 |
| U-Intracellular trafficking, secretion, and vesicular transport | 82 | 1.41 |
| V-Defense mechanisms | 72 | 1.24 |
| **Information storage and processing** |  |  |
| A-RNA processing and modification | 1 | 0.02 |
| B-Chromatin structure and dynamics | 1 | 0.02 |
| K-Transcription | 412 | 7.09 |
| L-Replication, recombination and repair | 226 | 3.89 |
| **Poorly characterized** |  |  |
| R-General function prediction only | 590 | 10.15 |
| S-Function unknown | 368 | 6.33 |
