# Supplementary material for: Complete Genomic Analysis of a Kingdom-Crossing Klebsiella variicola Isolate
Source: Front Microbiol. 2018 Oct 9;9:2428. doi: 10.3389/fmicb.2018.02428 (PMC6189331; doi:10.3389/fmicb.2018.02428)
Supplement: Supplementary file 8 [file Data_Sheet_1.docx]

Supplementary Material

**Complete Genomic Analysis of a** **Kingdom-Crossing**

***Klebsiella variicola* Isolate**

Yatao Guo*, Yao Zhai, Zhao Zhang, Daixi Li，Zhanwei Wang, Jingquan Li, Zilong He, Songnian Hu^5^, Yu Kang, Zhancheng Gao

*** Correspondence:** Zhancheng Gao, zcgao@bjmu.edu.cn

Yu Kang , kangy@big.ac.cn


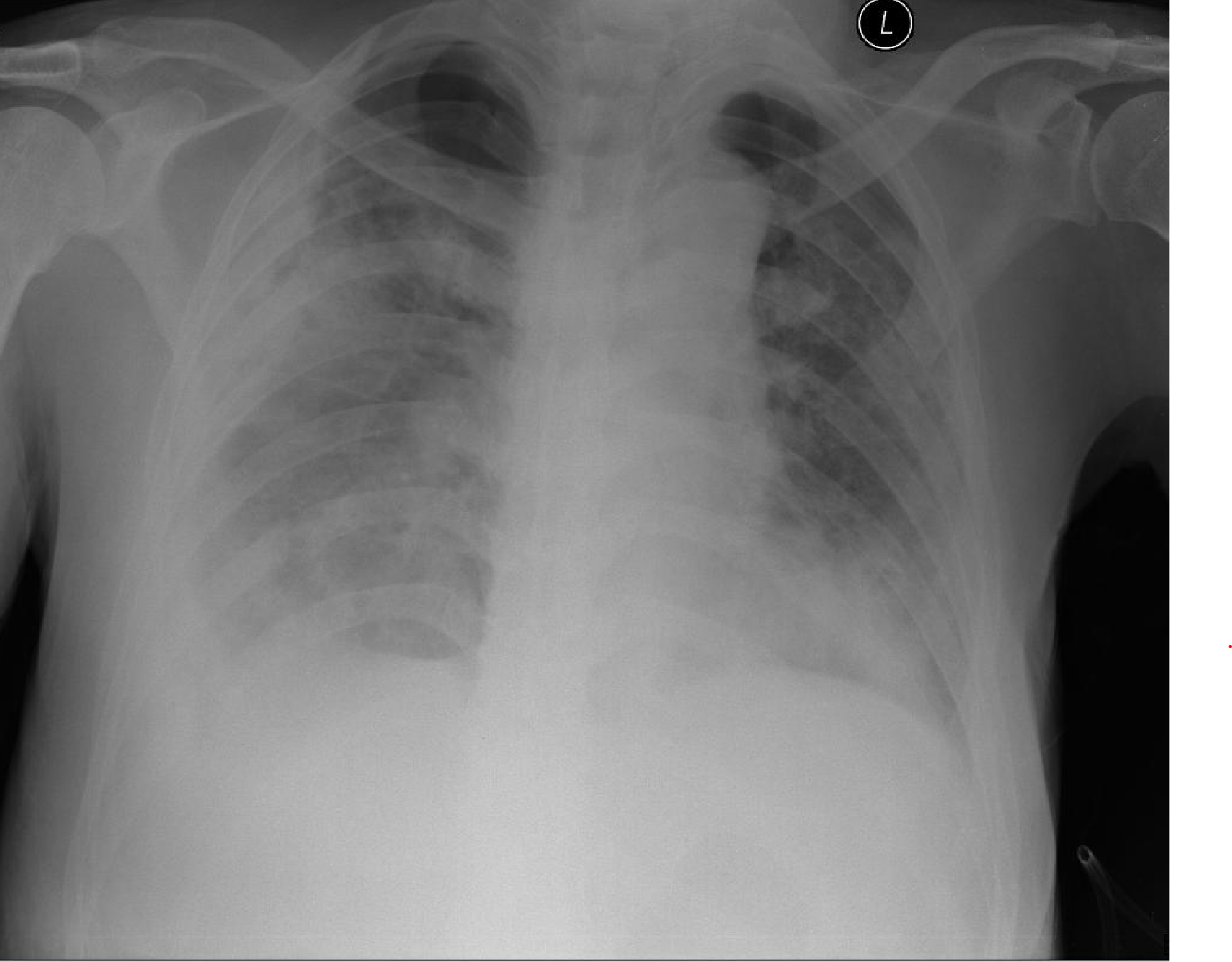


**Fig S1.** This chest radiograph was taken on the day that the patient died. It suggested that the lungs were scattered in high-density patches and the right pleural effusion.

**
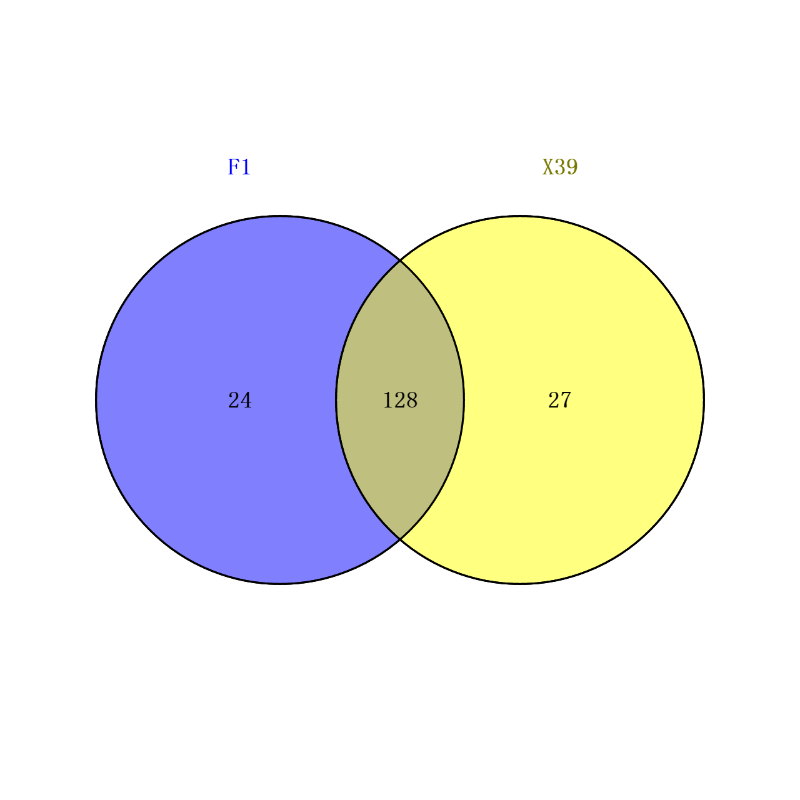
**

**Fig S2.** The Venn diagram of virulence factors of *K. variicola* X39 and *K. pneumoniae* F1.
